# Histopathologic analysis of gingival lesions: A 20-year retrospective study at one academic dental center

**DOI:** 10.4317/jced.54766

**Published:** 2018-06-01

**Authors:** Jazia A. Alblowi, Nada O. Binmadi

**Affiliations:** 1 BDS, DSc, DABP, Assistant Professor, Periodontology Department, King Abdulaziz University, Faculty of Dentistry, Jeddah, Saudi Arabia; 2 BDS, MBA, PhD, Assistant Professor, Oral Diagnostic Sciences Department, King Abdulaziz University, Faculty of Dentistry, Jeddah, Saudi Arabia

## Abstract

**Background:**

The gingiva is part of the periodontium supporting structures surrounding the teeth and commonly involved in gingival and periodontal conditions. Assessing the distribution of gingival lesions is important for evaluating the prevalence of periodontal disease in the population to optimize the oral health care services. The purpose of this study is to report the frequency and distribution of gingival lesions biopsied from 1996–2016.

**Material and Methods:**

This cross-sectional retrospective study retrieved data from all gingival lesions biopsied from 1996–2016 and sent to the King Abdulaziz University Dental Hospital oral pathology laboratory. Histologic sections were reviewed in a blinded manner by a certified oral pathologist to confirm the initial histologic diagnosis.

**Results:**

Of the 1,248 oral-maxillofacial lesions, 119 (9.5%) gingival lesions were diagnosed. The mean age was 41.58 years. Gingival lesions were more prevalent in female patients than male patients (53.8%). The most common diagnoses were reactive lesions (41.2%). Pyogenic granuloma was the predominant lesion in the category (n=26, 21.8%), and followed by inflammatory conditions (24.4%), benign neoplasm (9.2%), malignant neoplasm (7.6%), epithelial lesions (7.6%), miscellaneous (5%), and immune-mediated diseases (5%). Squamous cell carcinoma was the only malignant neoplasm reported (7.6%; mean age, 57.44 years) and more common in male than female patients (2:1). Most biopsies were sent from oral and maxillofacial surgeons (55.6%) followed by general dentists (22.2%) and periodontists (12.8%).

**Conclusions:**

Pyogenic granuloma was the most common gingival lesion. Squamous cell carcinoma was the only malignant lesion in which histologic examination was the definitive diagnostic measure. This study provides information about the frequencies and distributions of gingival lesions over 20 years.

** Key words:**Gingival biopsies, retrospective, reactive lesions, oral pathology.

## Introduction

Gingiva is a part of the periodontium-supporting structures surrounding the teeth and commonly involved in gingival and periodontal diseases. It can be subjected to trauma or irritation. It is a common site for various pathologic diseases (1,2). Despite the fact that gingivitis is the most common disease involving the gingiva, many other uncommon local or systemic pathologic conditions also may involve the gingiva, which necessitates that periodontists and pathologists work together to reach the appropriate diagnosis to ensure timely diagnosis and management (3).

Although diagnoses of gingival diseases and conditions depend on the clinical features, histologic examination might be needed to confirm the diagnosis as well as to rule out a neoplastic nature of the process. Only a few epidemiologic studies were reported in the literature regarding gingival lesions (2,4-8).

In Saudi societies, little is known about the frequencies of different oral lesions as confirmed by histologic diagnosis. Most of these studies investigated oral lesions in general and indicated the prevalence of malignancies in different areas of the oral cavity (6,7). The prevalence of lesions might vary in different countries and geographic locations and the histologically confirmed diagnoses of gingival lesions are not well reported in Saudi Arabia. The purpose of this study is to evaluate the frequencies and distributions of gingival lesions biopsied at King Abdulaziz University Dental Hospital during the last 20 years from 1996-2016 diagnosed by clinical and histologic features.

## Material and Methods

This retrospective cross-sectional study was approved by the Research Ethics Committee, Faculty of Dentistry, King Abdulaziz University (protocol number: 088-16). All gingival biopsies (n=119) sent to the Oral and Maxillofacial Pathology (OMFP) laboratory at King Abdulaziz University, Jeddah, Saudi Arabia from different departments in the Faculty of Dentistry at King Abdulaziz University Dental Hospital during the period from 1996–2016 were evaluated and the corresponding medical records were reviewed. Cases with inadequate histories or reports (n=7) were not included in the sample. The archived demographic data concerning age and sex were retrieved. The referring dentists and specialists were also recorded. All patients’ identifiers were removed and the samples were given unique identifiers. The location and type of the gingival lesions were obtained from the register of the pathology laboratory and clinical information concerning the lesion was retrieved from the corresponding biopsy request form. The archived 5-µm-thick paraffin sections, which were stained previously with hematoxylin and eosin, were reviewed by a certified oral pathologist to confirm the initial histologic diagnosis in a blinded manner. The lesions were classified into groups as follows: reactive/adaptive lesions, inflammatory lesions, benign neoplasms, malignant neoplasms, epithelial lesions, immune-mediated diseases (IMD), and miscellaneous.

Statistical analysis was performed using the Statistical Package for the Social Sciences (SPSS) version 22 (IBM Corp., Armonk, NY, USA). Categorical variables are presented as numbers, nominal variables as percentages, and continuous variables as means and standard deviations.

## Results

Over the 20-year duration (1996-2016), 119 gingival tissue samples were diagnosed out of the total of 1,248 oral-maxillofacial lesions, so the incidence of gingival lesions was 9.5%. Of the 119 patients, 64 of were female (53.8%) and 55 patients were male (46.2%) with a mean age of 41.58 years (range, 6-84 years). The youngest, a 6-year-old male child, had a myofibroma, and the oldest, an 84-year-old man, had mild dysplasia.

The most frequent histopathologic category along with age and sex distributions is summarized in  Table 1. The most common diagnoses were reactive lesions, which accounted for 41.2%, followed by inflammatory lesions (24.4%), benign neoplasms (8.4%), malignant neoplasms (7.6%), epithelial lesions (7.6%), miscellaneous (5.9%), and IMD (5%).

**Table 1:**
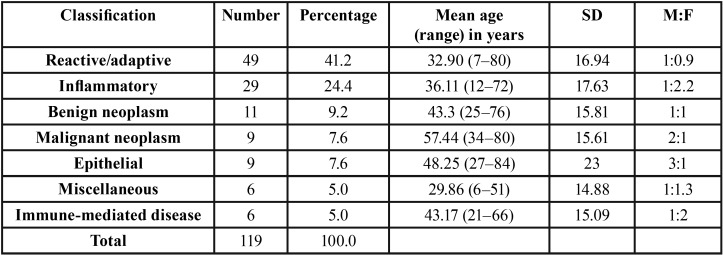
Frequencies of different histopathologic categories with age and sex distributions.

**Figure 1:**
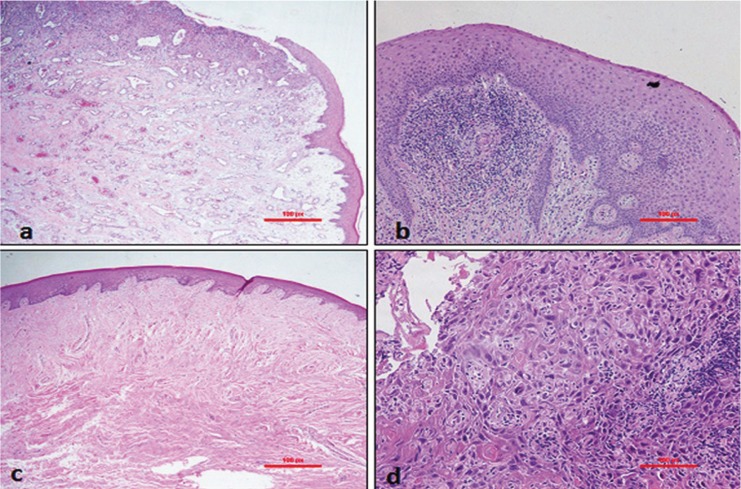
Histopathologic images of (a) a pyogenic granuloma showing ulceration of the oral epithelium with proliferation of new blood vessels and chronic inflammation are shown -H&E ×4; (b) gingival tissue shows focal diffuse infiltration of chronic inflammatory cells (chronic gingivitis) -H&E ×4; (c) a fibroma with thickened collagen bundles around the vessels in the lamina propria- H&E ×10; and (d) squamous cell carcinoma shows malignant cellular and architectural changes such as hyperchromatism, polymorphism, and keratin pearls - H&E ×20.

 Table 2 shows the frequency of each histopathologic diagnosis along with age and sex distributions. The most common reactive/adaptive lesion in the gingival tissue was pyogenic granuloma (n=26, 21.8%) (Fig. 1a), which was more common in male patients (n=15) with a mean age of 32.12±15.21 years. The most frequent inflammatory lesion was chronic gingivitis (n=22, 18.48%) (Fig. 1b) and was more common in female patients (n=14) with a mean age of 36.62±19.54 years. Fibroma was the predominant benign neoplasm (n=11, 9.2%) (Fig. 1c) and was equally distributed among male and female patients with a mean age of 43.30±15.81 years. Oral squamous cell carcinoma (OSCC) was the only malignant neoplasm reported in gingival tissue among our specimens (n=9, 7.6%) (Fig. 1d) with a mean age of 57.44 years; it showed a male predominance (male-to-female ratio: 2:1). Hyperkeratosis and acanthosis (n=7, 5.88%) were the most commonly observed histopathologic findings among epithelial lesions and were observed more frequently in male patients with a mean age of 42±20.9 years. Among immune-mediated lesions, lichen planus (n=4, 3.36%) was the most common with a 1:1 male-to-female ratio and mean age of 47.5±13.53 years. Miscellaneous cases such as metal pigmentation, intramucosal nevus, and normal tissue accounted for 5% of the total number of gingival specimens.

**Table 2:**
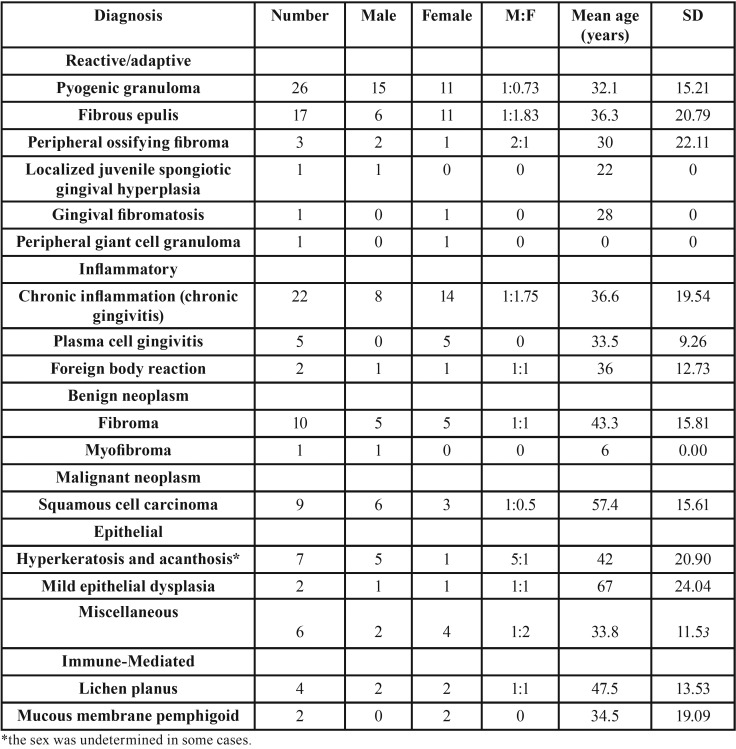
Age and sex distributions for each histopathologic diagnosis.

**Figure 2:**
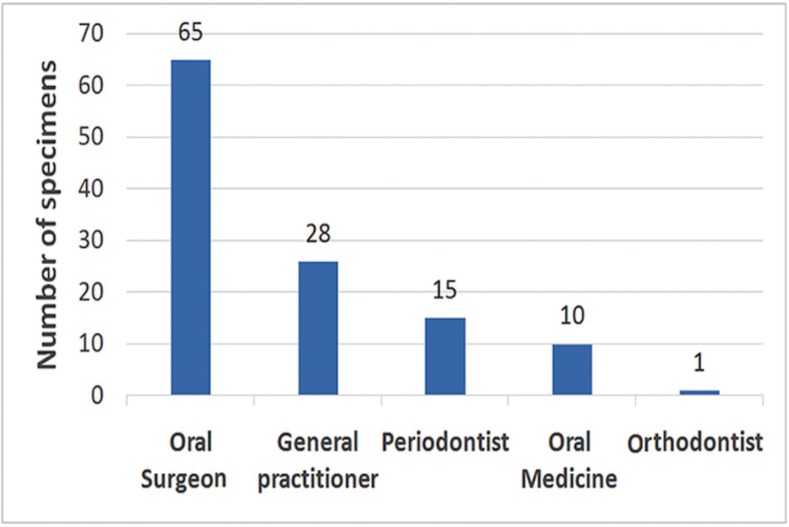
Distribution of the specimens’ referral sources by specialty.

The dental specialty referral pattern for gingival specimens was distributed as follows: oral and maxillofacial surgeons, 54.6% (n = 65); general dentists, 23.2% (n = 28); periodontists, 12.6% (n = 15); oral medicine practitioners, 8.4% (n = 10); and orthodontists, 0.8% (n = 1) (Fig. 2).

## Discussion

To the best of our knowledge, the present study represents first attempt to report on the frequency and distribution of the gingival lesions biopsied at King Abdulaziz University Dental Hospital during the last 20 years from 1996–2016 diagnosed on the basis of clinical and histologic features.

The total number of gingival lesions (n=119) accounted for 9.5% of all cases analyzed by the laboratory. Most gingival lesions in this study were from the middle-age group with a mean age of 41.58 years, which was older than the ages in studies performed by Kamath *et al.* and Shamim *et al.* (5,8). The gingival lesions were predominant in female patients (53.8%) compared to male patients (46.2%); which was consistent with the findings of other reported retrospective studies (5,8-11). Although non-neoplastic lesions were prevalent in females, neoplastic lesions were more prevalent in males.

The majority of samples were found to be non-neoplastic lesions. The most common gingival lesions were reactive/adaptive lesions (41.2%) ( Table 1) with more prevalence in male patients than females and in agreement with the earlier report by Kamath *et al.* (5). Pyogenic granuloma was the most common lesion in this category. The results were similar to earlier reports (8-10). Pyogenic granuloma represented more than one-fifth of all gingival lesions with a peak incidence of occurrence at age 32.1 years, which was slightly older than earlier reports (8). It is more prevalent in males than females of our study population which is in contrast to earlier reports which showed female prevalence (8-12). Pyogenic granuloma was clinically characterized as a gingival lump that bled easily and demonstrated surface ulceration (12,13).

The second most common gingival lesion was inflammatory conditions and chronic inflammation was the most frequent histologic finding. Gingivitis is a common form of the inflammatory disease that results as a response to plaque accumulation (14). Gingivitis was prevalent in female patients with a mean age of 36.6 years, which was older than the age reported by Ababneh (10).

Fibroma was the most common benign tumour, accounting for 8.4% (n=10) of the gingival lesions and is in agreement with earlier reports (5,8). It was distributed equally among males and females. The peak incidence of occurrence was at the age of 43.3 years, similar to the results of the study performed by Shamim *et al.* (8).

Malignant neoplasms of the gingiva accounted for 7.6% of the total number of cases, which was higher than reported in the study by Manjunatha *et al.* (9) but lower than reported in other epidemiologic data (5,8,10) and is in accordance with reports by Mario Carbone *et al.* (11). OSCC was the only malignant neoplasm reported in gingival biopsy specimens in this study, which was in accordance with the results of many other studies (5,8,11). The gingiva is considered one of the most common sites for OSCC (11). The mean age of occurrence of OSCC in our study was 57.4 years and was more common in males, which is an agreement with the results of Kamath *et al.* and Ababneh (5,10).

The epithelial lesions represented 7.6% (n=9) of the biopsied gingival lesions. Mild dysplasia accounted for 22 % (n = 2) of epithelial lesions. The prevalence is higher than that found by Carbone and coworkers (11). IMD can present clinically in the gingiva as an ulcer, vesicles, or erosive lesions and is called desquamative gingivitis. The most common IMD in gingival was pemphigoid, which differed from our findings that showed oral lichen planus as the most common lesion in this category and is in agreement with reports by Mario Carbone *et al.* (11).

Almost all users of the gingival biopsy and histopathology services at the school were dental specialists; only 22.2% were general dentists. Most specimens were submitted by oral and maxillofacial surgeons followed by periodontists and oral medicine specialists. This result is different from that in the study by Wan *et al.* where oral medicine specialists submitted the most oral biopsies in Australia (15). Haberland *et al.* showed that most oral pathology referrals were from general dentists followed by periodontists (16). We think that most of oral lesions cases were referred to oral surgeons for management rather than oral medicine specialists because of unrecognition and loss of identity of this specialty in non-academic sectors in the country.

One of the limitations of this study is the sample size, which could be explained in part by the fact that this study was conducted at only one academic centre. Moreover, we were unable to evaluate risk factors such as socioeconomic status, occupation, and oral habits in this study, which were usually not mentioned on the requisition forms.

In conclusion, the current data can be useful in updating information regarding the prevalence and characteristics of gingival lesions in this region. This is crucial, as it shows that clinicians in general and periodontists in specific were most of the gingival lesions referred to, the types of lesions that they might expect to encounter in their practices. It also serves as baseline data for future prevalence studies on gingival lesions in Jeddah. Further multicenter studies are encouraged to better represent the epidemiologic findings of gingival lesions in the Saudi population.
